# Event-related potentials study on the effects of high neuroticism on senile false memory

**DOI:** 10.1371/journal.pone.0304646

**Published:** 2024-08-15

**Authors:** Wenju Zhang, Yibin Zhou, Yan Zhang, Xianghong Zhan

**Affiliations:** 1 Traditional Chinese Medicine School, Henan University of Chinese Medicine, Zhengzhou, Henan, China; 2 Henan Provincial Engineering Technology Research Center for Chinese and Western Integrative Prevention and Treatment of Brain Cognitive Disease, Zhengzhou, Henan, China; 3 Zhengzhou Key Laboratory for Chinese & Western Integrative Prevention and Treatment of Brain Cognitive Disease, Zhengzhou, Henan, China; 4 School of Medicine, Henan University of Chinese Medicine, Zhengzhou, Henan, China; La Sapienza University of Rome, ITALY

## Abstract

**Objective:**

To study the false memory among senile normal people with high neuroticism and low neuroticism using neuropsychological scales and event-related potentials (ERPs), and to explore the effects of high neuroticism on false memory and its neuroelectrophysiological mechanism.

**Methods:**

A cross-sectional study was conducted, in which the general situation questionnaire, adult version of Eysenck personality questionnaire (EPQ) and Montreal cognitive assessment (MoCA) scale were used to establish a multi-dimensional survey in senile normal people over 60 years old from communities in Zhengzhou, and the EPQ and general situation questionnaire were used to comprehensively screen and divide the study subjects into high neuroticism group and low neuroticism group from 206 senile people. The population was matched by 1:1 according to gender, age (±2 years), and years of education (±2 years), and 40 subjects were finally enrolled for detection of electroencephalograph (EEG) components using ERPs. The Deese-Roediger-McDermott (DRM) paradigm of false memory was designed using E-prime2.0 system, and the stimulus program was presented. The EEG signals of the study subjects were recorded in real time and acquired using 64-channel Neuroscan EEG signals acquisition system.

**Results:**

High neuroticism group was evidently lower in the mean accuracy than low neuroticism group, and the difference in the mean accuracy was statistically significant (*P* = 0.013), but the difference in reaction time was not statistically significant. 2. The mean amplitude of EEG component N400: The difference in the main effect of N400 in the brain region was significantly different (*P*<0.001), and the mean amplitude of N400 was the largest in frontal region, followed by central region and parietal region successively (all *P*<0.05). There was no statistically significant difference in the neurotic main effect or the interaction effect of neuroticism and brain region. The latency of N400: There was no significant difference in the neurotic main effect, main effect of the brain region or the interaction effect of neuroticism and brain region. 3. The mean amplitude of EEG component LPC: The difference in the main effect of the brain region was significantly different (*P<*0.001), and the mean amplitude of LPC was the largest in frontal region, followed by central region and parietal region successively (all *P*<0.05). There was no significant difference in the neurotic main effect, neuroticism or the interaction effect of neuroticism and brain region. As to the latency of LPC, there was significant difference in the main effect of the brain region (*P* = 0.025), and the latency of LPC was shorter in frontal region than that in central region (*P*<0.05). The differences in the neurotic main effect, interaction effect of neuroticism and brain region were not statistically significant.

**Conclusions:**

High neuroticism can significantly increase the false memory of senile normal people. The EEG components N400 and LPC are potential early indicators of high neuroticism affecting false memory. High neuroticism may influence false memory by affecting the frontal cortex function.

## Introduction

With the economic, social and medical development, people’s life expectancy and the number of senile people are increasing universally, and the ageing problem of the population is gradually aggravated. With prolonged life span, the body function is deteriorating in the ageing process, and the incidence of cognitive dysfunction mainly manifested as memory decline is also increasing, which not only brings about heavy medical pressure and economic burden to the patients, families and society, but also seriously affects the quality of life of those patients. Therefore, the decline of memory function has become a focus of attention and research.

At present, the cognitive psychology technology applied to study the cognitive function is mainly in the memory field [1]. Through the experimental studies of cognitive function, we have gradually explored the mechanisms of cognitive decline [2]. In life, people sometimes falsely recall events that never happened, or recall events that were not exactly the same as their actual experiences. This common phenomenon is false memory. The ageing effect of false memory refers to the phenomenon that elderly people are more likely to have false memories than young people under the same situation and conditions. Devitt et al. [3] found that people’s sensitivity to false memory increases with age, and the aging effect of false memory may be related to the function decline of some regions in the frontal lobe and medial temporal lobe, resulting in impairment of correlation and monitoring ability. Michelle et al. [4] found that healthy senile people showed decreased frontal lobe function due to normal ageing. Healthy senile people are more likely to have false memories than healthy young people. Katja Volz et al [5, 6] found that such EEG components as N400, LPC were closely related to memory-based processes in the course of recognition. All the above studies suggest that false memory reflects human cognitive function and is closely related to age. False memory may be an early sensitive indicator of cognitive decline in the ageing process, and the study on the effects of high neuroticism on false memory is of great significance for the research on ageing.

Modern studies have shown that people with high neuroticism are prone to experience chronic emotional stress, causing excessive activation of the hypothalamic-pituitary-adrenal axis (HPA axis), and neuronal damage caused by a variety of inflammatory factors occur mostly in the hippocampus and prefrontal cortex. It is generally believed that the prefrontal cortex is associated with individual false memory, participates in the encoding and extraction of memory, and plays an important role in monitoring. It is very likely that individuals with high neuroticism will have false memory due to prefrontal cortex injury, which will lead to a decline in overall cognitive function. The study has found that emotions have an important influence on individual’s false memory. The author believes that high neuroticism can significantly increase the false memory of senile people.

## Study subjects and methods

### Study subjects

The study subjects were volunteers of senile normal people from the communities in Zhengzhou recruited by the research group. The recruitment time range of the study subjects was from June 5, 2022 to September 30, 2022. The subjects were required to voluntarily accept the neuropsychological scale and ERPs detection. They did not suffer from diseases that significantly affected cognitive function, and their visual and auditory functions were normal and met the experimental requirements. The subjects’ written consent was obtained for this study. The protocol was reviewed and approved by the Ethics Committee of the First Affiliated Hospital of Henan University of Chinese Medicine (Ethical number: 2019HL-136-01).

#### Inclusion criteria

Senile normal people over 60 years old, Chinese Han nationality, Chinese as mother language, right-handed, junior middle school education or above; normal visual, auditory and language functions; normal daily living ability.

#### Exclusion criteria

Subjects who did not cooperate with investigation and tests; subjects with lesions of important organs (heart, liver, spleen, lung, or kidney); subjects with dementia, epilepsy, Parkinsons, stroke or other neurological disease affecting cognitive function; subjects with severe mental disorder (such as depression syndrome, and anxiety syndrome,), colour blindness, or severe colour weakness; subjects with a history of severe head trauma accompanied by cognitive impairment or abnormal brain structure and function.

#### Rejection criteria

Subjects with severe EEG drift; subjects with incomplete data; subjects who did not complete the study.

#### Grouping of the experiment

According to the EPQ adult scale score of the subjects, the normal people who met the inclusion criteria were divided into high neuroticism group if the N-scale conversion score of the EPQ scale was > 61.5 points and low neuroticism group if the N-scale conversion score of the EPQ scale was < 38.5 points. After all tests such as the scale and ERPs were completed, the subjects could receive a certain amount of compensation of labor-hour lost.

### Study methods

A cross-sectional study was conducted, in which the general situation questionnaire, adult version of Eysenck personality questionnaire (EPQ) and Montreal cognitive assessment (MoCA) scale were used to establish a multi-dimensional survey in senile normal people over 60 years old from communities in Zhengzhou, and the EPQ and general situation questionnaire were used to comprehensively screen and divide the study subjects into high neuroticism group and low neuroticism group from 206 senile people. The population was matched by 1:1 according to gender, age (±2 years), and years of education (±2 years), and 40 subjects were finally enrolled for detection of EEG components using ERPs. The number of ERPs samples in each group was 20.

#### Experimental instruments

This study relied on the Henan Provincial Engineering Technology Research Center for Chinese and Western Integrative Prevention and Treatment of Brain Cognitive Disease and 64-channel ERPs workstation of Zhengzhou Key Laboratory for Chinese and Western Integrative Prevention and Treatment of Brain Cognitive Disease where the research group was located. The DRM paradigm of false memory was designed using E-prime2.0 system, and the stimulus program was presented. The EEG signals of the study subjects were conducted real-time recording and acquisition using 64-channel Neuroscan EEG signals acquisition system.

#### Experimental materials

In this study, the classical DRM paradigm experimental materials [7] were used and appropriately modified. There were 12 Chinese word lists to be learned and 12 words in each list; each word was associated with the key lure word in the word list. The words were presented in order of the degree of association with the key lure word from high to low. There were 144 words to be learned in total. The recognition word lists consisted of 108 words from three parts, of which 48 words had been learned and were selected from the first, fifth, eighth and eleventh ones respectively of the 12 learned word lists; 12 key lure words from 12 learned word lists; 48 unrelated words selected from the first, fifth, eighth and eleventh ones respectively of the other 12 unlearned word lists. In the recognition test, all 108 words were presented as pseudo-random. In order to avoid proximity effects, another 3 unrelated words always appeared in a fixed order at the beginning of the test, and subjects did not analyze their reaction data.

#### Experimental procedure

**Fig 1:**
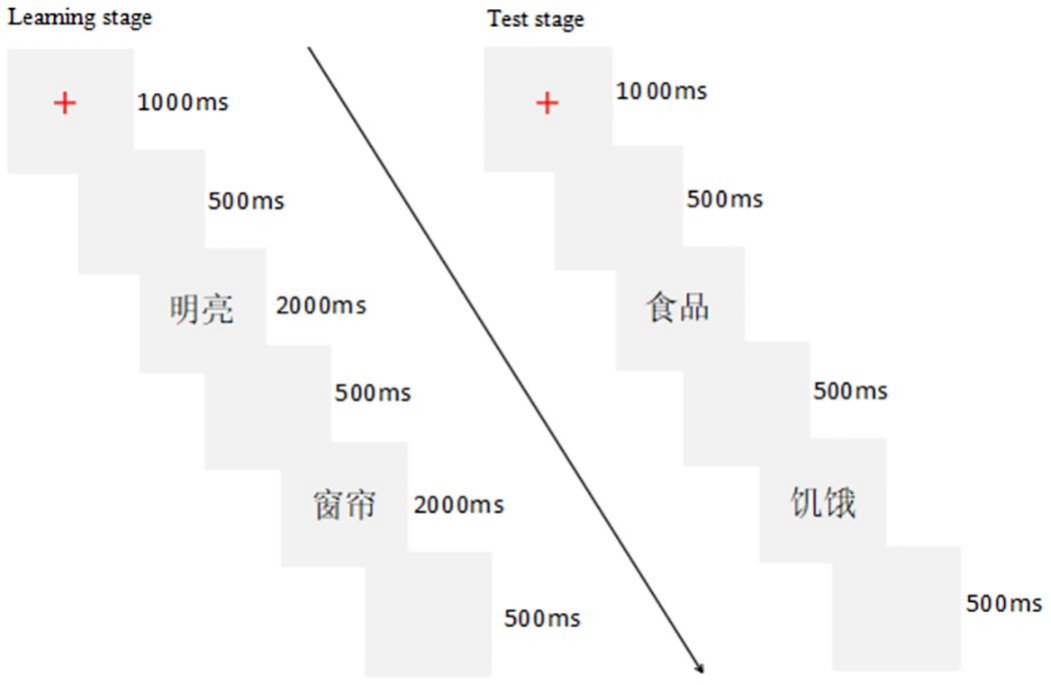
Flow chart of DRM paradigm. “明亮” in the upper left means “bright”, “窗帘” in the lower left means “curtain”, “食品” in the upper right means “food”, and “饥饿” in the lower right means “hunger”.

The experimental program was written using the E-prime 2.0 system. The experiment process consisted of two stages: learning stage and testing stage, both of which were completed on the computer. In the learning stage, subjects were asked to learn words presented in groups of 12 in turn, with a red “+” symbol between each group to signal the start of a new group. A total of 12 groups were presented. After preparation, subjects pressed the “Q” key to begin learning. Once this key was pressed, a red “+” symbol appeared in the center of the computer screen for 1000ms, followed by a blank screen for 500ms. Then, the words were presented one by one in black Song font of size 40, with each word displayed for 2000ms and followed by a blank screen for 500ms. After all 12 words in a word list were presented, another red “+” symbol appeared in the center of the screen for 1000ms, followed by a blank screen and the presentation of the next group of words. After all the words had been learned, the experiment entered the testing stage. In the testing stage, if the subject thinks that the words presented on the screen are just learned, press the "F" key; if the subject thinks it is something they have not learned before, press the "J" key. If the subject can’t remember, he can guess by feeling, but he can’t omit not to answer. Ask the subject to remember the choice represented by each key, and press the "Q" key when ready to start the test. The test words are presented in sequence in the center of the computer screen, and the font, size, and color of the words are the same as in the learning stage. After the subjects pressed the button to make a judgment, the word disappeared, and then the next word was presented. After all the test words were presented, the experiment ended. In the test stage, the "F" and "J" response keys were balanced between the left and right hands. The flow chart of DRM paradigm is shown in Fig 1.

#### EEG recording

ERPs detection was conducted in the standardized sound insulation laboratory of Henan Provincial Engineering Technology Research Center for Chinese and Western Integrative Prevention and Treatment of Brain Cognitive Disease and 64-channel ERPs workstation of Zhengzhou Key Laboratory for Chinese and Western Integrative Prevention and Treatment of Brain Cognitive Disease. Before the EEG test, the experimenter first helped the subject to familiarize themselves with the experimental environment and the testing process to eliminate neuroticism, unfamiliarity or other unrelated emotional factors that may affect the experiment. After washing and drying the hair, the experimenter put a 64-channel Neuroscan adaptive Ag/AgCl electrode cap on the subject and used imported Quik-Gel electrode paste to reduce the scalp resistance of the subject to below 5kΩ. Finally, the test procedure should be started after the subject was emotionally stable and fully relaxed. The whole experiment process took about 60 minutes. After the experiment, the subjects were given a certain amount of labor-hour lost compensation.

EEG information collection using ERPs: The E-prime2.0 system was applied to present the DRM paradigm stimulus program for false memory, and the Curry8 acquisition system of Neuroscan equipment and SynAmps 64-channel amplifier box were used to record and transmit EEG signals. The electrode sites distribution of the 64-channel caps used complied with the internationally accepted 10–20 extended electrode system, and the EEG data of the following 64 scalp electrode sites were finally recorded (FP1, FPZ, FP2, AF3, AF4, F7, F5, F3, F1, FZ, F2, F4, F6, F8, FT7, FC5, FC3, FC1, FCZ, FC2, FC4, FC6, FT8, T7, C5, C3, C1, CZ, C2, C4, C6, T8, TP7, CP5, CP3, CP1, CPZ, CP2, CP4, CP6, TP8, P7, P5, P3, P1, PZ, P2, P4, P6, P8, PO7, PO5, PO3, POZ, PO4, PO6, PO8, CB1, O1, OZ, O2, CB2, M1, and M2). Electrodes were placed at the intersection of the lateral sides of the lateral canthus of the eyes and the lower vertical line of the lateral side of the eyebrow arch to record the horizontal electrooculograph (HEOG), and electrodes were placed at 0.5cm above and below the left orbit to record the vertical electrooculograph (VEOG). Take the mean EEG values of bilateral mastoid M1 and M2 as reference, set the sampling rate to 1000Hz, DC-200Hz to the recording strip, continuously maintain the scalp impedance of each electrode below 5kΩ throughout the testing process, and synchronously record the continuous EEG data and behavioural data.

#### ERPs data processing

In this study, Matlab 2013b software was used for offline analysis of the collected ERPs data. First, the collected ERPs data were segmented: the segment timing was selected from 200ms before the presentation of the target stimulus to 1000ms after the presentation of the target stimulus (-200ms to 1000ms). Then, the obtained data were baseline corrected: the baseline was selected from 200ms before the presentation of the target stimulus to 0ms (-200ms to 0ms) for baseline correction. Subsequently, the data underwent filtering operation: setting bandpass filtering at 0.5-30Hz and notch filtering at 48-52Hz, and filtering the data offline, followed by artifact rejection: correcting artifacts of horizontal and vertical eye movements respectively, and excluding artifact signals with amplitudes greater than±100μV. The behavioral responses of each subject under the false memory DRM paradigm task condition were marked as correct ERPs waveform segmented data, and the data were superimposed and averaged, and the total mean ERPs waveform of the subjects in each group were finally obtained.

### Statistical analysis

SPSS22.0 statistical analysis software was applied for data management and analysis. Quantitative data were described as mean ± standard deviation (M±SD), age and educational level were compared using the independent sample T test. Qualitative data were described using frequency indicators and compared with rank sum test. The independent sample T test was performed on the accuracy and reaction time of the DRM paradigm task for false memory in two kinds of neurotic populations. Based on relevant literature [8, 9] and the objective of this experiment, the whole brain topographic map of ERPs in each group and the total mean waveform of each electrode site, the mean amplitude and latency of the two main ERPs components of N400 and LPC were finally selected for analysis. Nine electrode sites of the frontal, central and parietal regions were selected for comparative analysis, namely the frontal region (F3, FZ, F4), central region (C3, CZ, C4) and parietal region (P3, PZ, P4). The mean amplitude and latency of the following time windows were compared: 300-500ms for the N400 time window and 500-800ms for the LPC time window. Finally, the mixed analysis of variance of 2 (neuroticism: low neuroticism/high neuroticism) × 3 (brain region: frontal region/central region/parietal region) was conducted on the mean amplitude and latency of the two EEG components of N400 and LPC, the *P* value was corrected using the Greenhouse-Geisser method, and partial *η*^2^ was calculated as a measure of effect size. In the above statistical analysis, *P*<0.05 indicated that the difference was statistically significant.

## Results

### General information of the study subjects

Senile subjects with low neuroticism or high neuroticism were all 20 cases. The differences were not statistically significant in the following general information, as specified in Table 1.

**Table 1:** General information of the study subjects.

		Low neuroticism group	High neuroticism group	*t-value*	*p-value*
Mean age (years old)		66.20 ± 3.75	66.00 ± 3.93	−0.165	0.870
Mean years of education (years)		11.35 ± 2.54	11.60 ± 2.33	0.325	0.747
Gender	Males	5 (12.5%)	5 (12.5%)	-	-
	Females	15(37.5%)	15 (37.5%)	-	-

### DRM paradigm behavioural results of false memory

Independent sample T test was performed on the mean accuracy and reaction time for the study subjects in the memory recognition task. The results showed that high neuroticism group was significantly lower in the mean accuracy than low neuroticism group, and the difference in the mean accuracy was statistically significant (*t*(38) = 2.61, *P* = 0.013), but the difference in reaction time was not statistically significant, as specified in Table 2.

**Table 2:** Mean accuracy and reaction time in memory recognition task (M±SD).

	Low neuroticism group	High neuroticism group
Accuracy (%)	0.73 ± 0.05	0.69 ± 0.05
Reaction time (ms)	1076.57 ± 166.31	1037.70 ± 192.42

### DRM paradigm ERPs detection results of false memory

**Fig 2:**
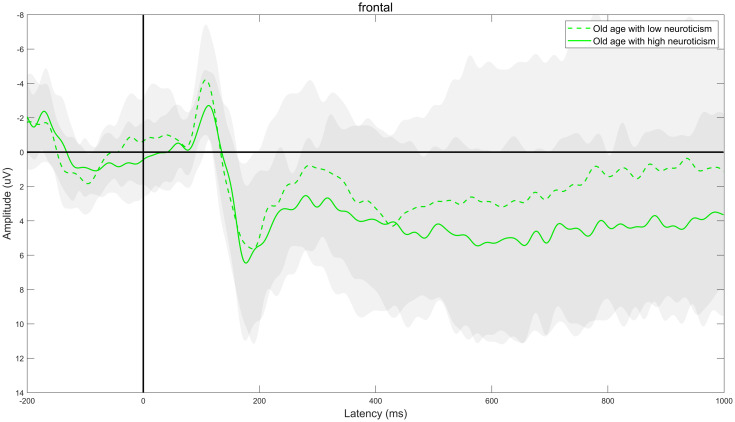
Total mean waveform of electrode sites in the frontal region. The green dashed line represents low neuroticism in senile people, while the green solid line represents high neuroticism in senile people. The gray shading represents the standard deviation distribution; the dark grey area represents the overlap of standard deviation distribution of high neuroticism and low neuroticism group, while the light gray area represents the non-overlapping part of standard deviation distribution of high neuroticism and low neuroticism group.

**Fig 3:**
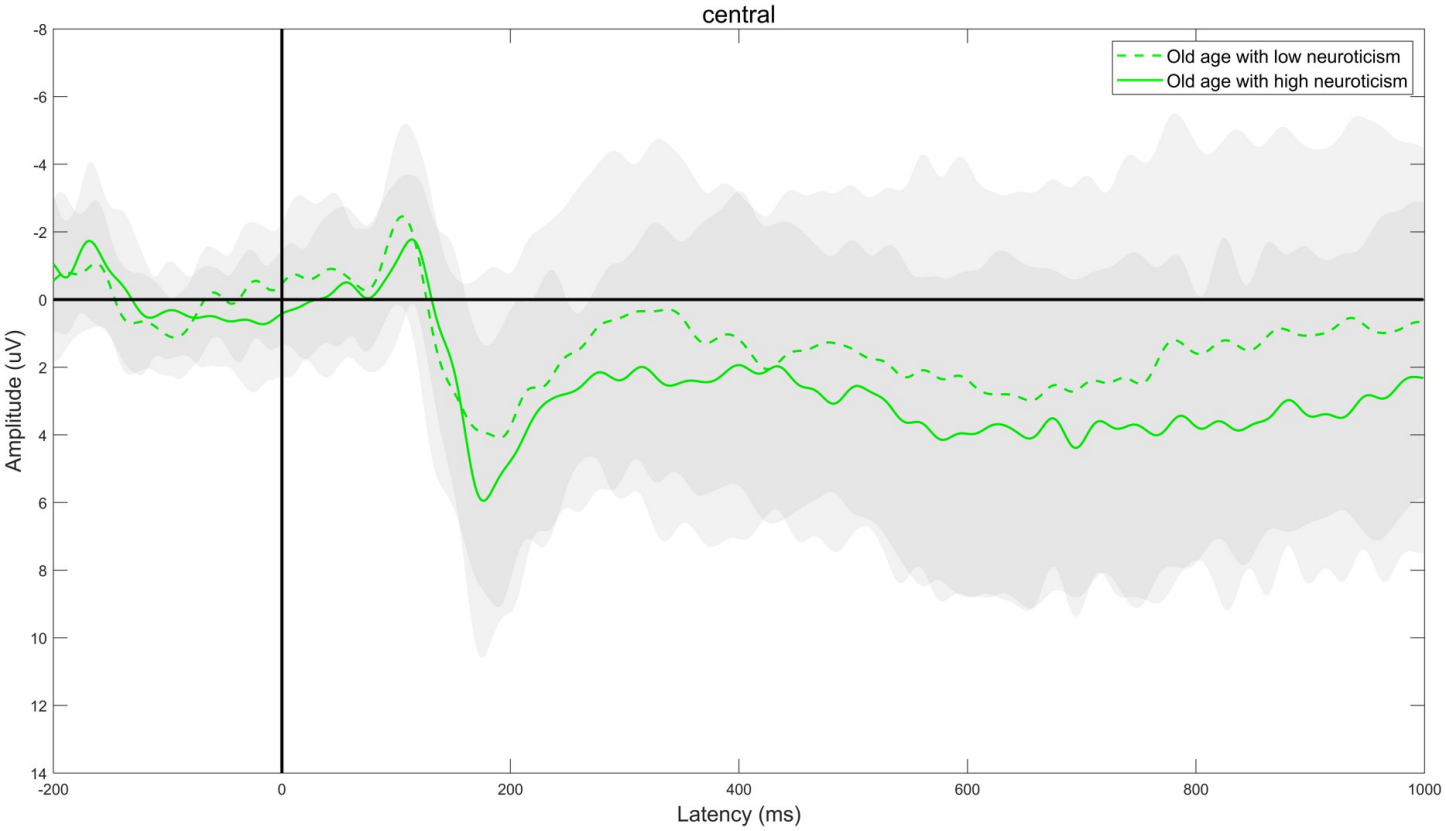
Total mean waveform of electrode sites in the central region. The green dashed line represents low neuroticism in senile people, while the green solid line represents high neuroticism in senile people. The gray shading represents the standard deviation distribution; the dark grey area represents the overlap of standard deviation distribution of high neuroticism and low neuroticism group, while the light gray area represents the non-overlapping part of standard deviation distribution of high neuroticism and low neuroticism group.

**Fig 4:**
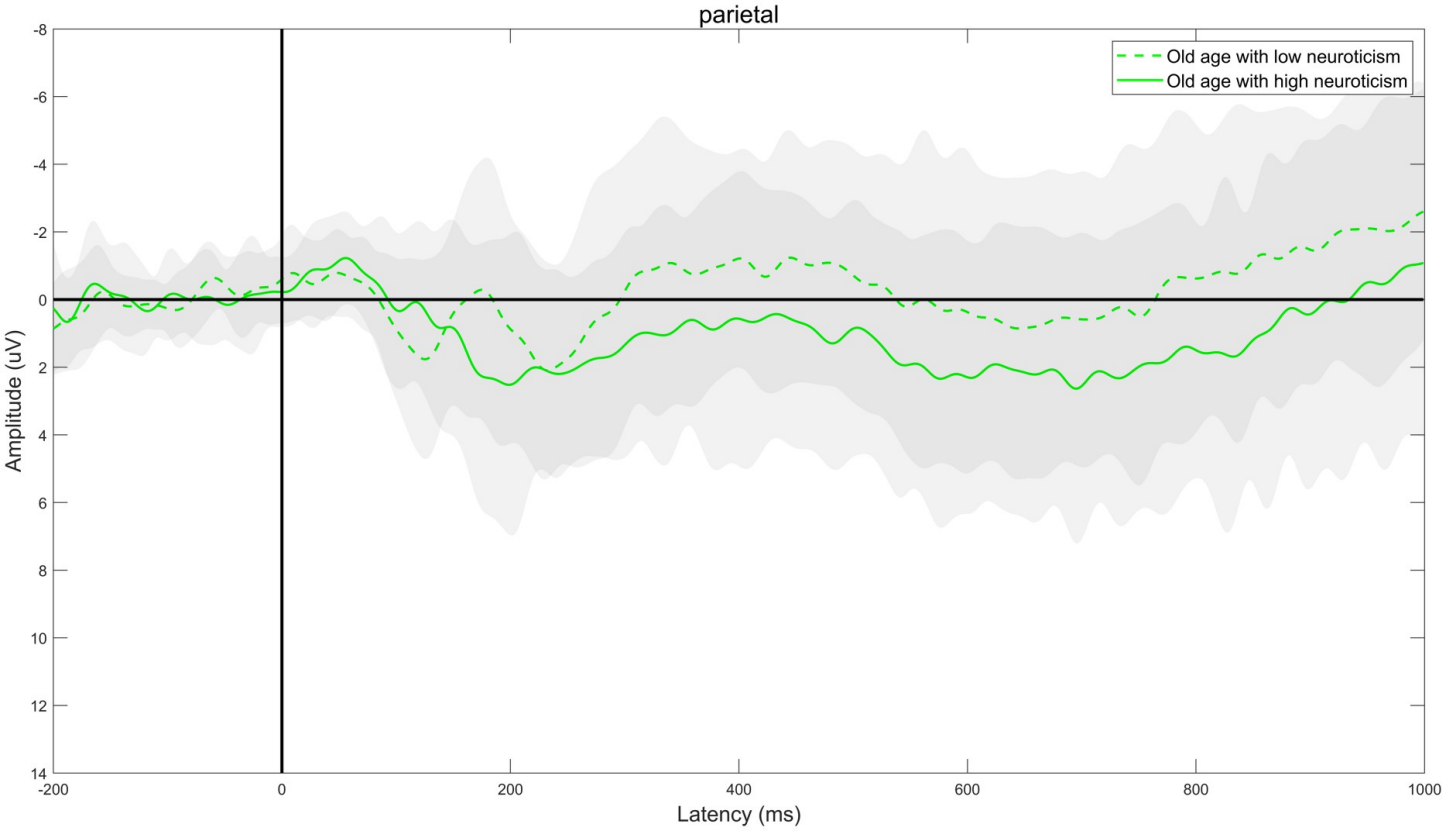
Total mean waveform of electrode sites in the parietal region. The green dashed line represents low neuroticism in senile people, while the green solid line represents high neuroticism in senile people. The gray shading represents the standard deviation distribution; the dark grey area represents the overlap of standard deviation distribution of high neuroticism and low neuroticism group, while the light gray area represents the non-overlapping part of standard deviation distribution of high neuroticism and low neuroticism group.

**Fig 5:**
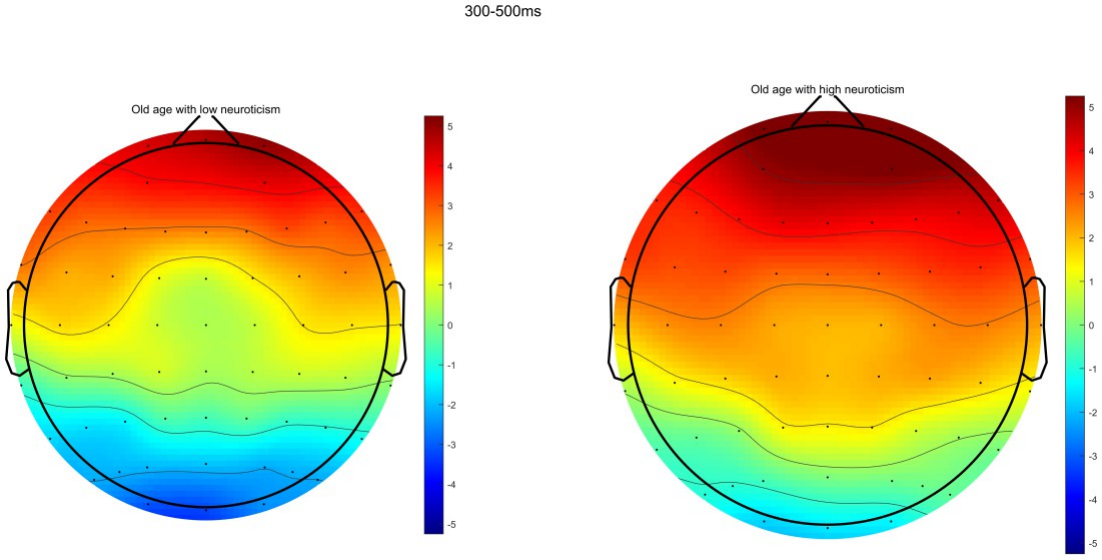
Brain topographic map of N400. The left represents low neuroticism in senile people, while the right represents high neuroticism in senile people. The unit of the color bars is μv.

**Fig 6:**
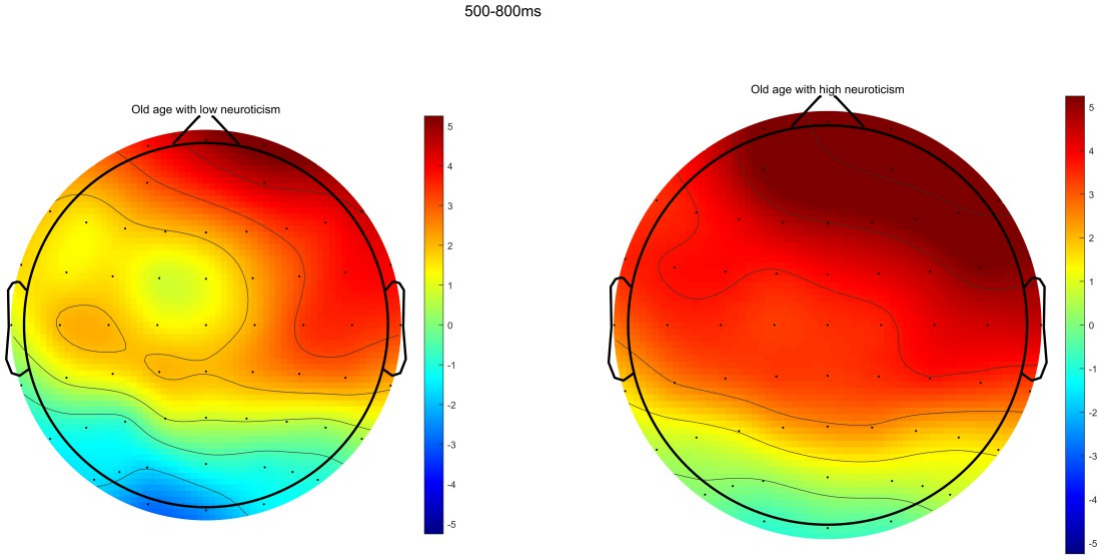
Brain topographic map of LPC. The left represents low neuroticism in senile people, while the right represents high neuroticism in senile people. The unit of the color bars is μv.

Through ERPs detection, it was found that the task of DRM paradigm of false memory obviously induced the EEG components of N400 and LPC. Specifically, the electrode sites of frontal region (F3, FZ, and F4), central region (C3, CZ, and C4) and parietal region (P3, PZ, and P4) were taken as examples. The comparison of the total mean waveform of electrode sites in each brain region is shown in Figs 2 to 4. Comparison of brain topographic map of EEG components of N400 and LPC is shown in Figs 5 and 6.

#### Mean amplitude of EEG component N400

The difference in the main effect in the brain region was statistically significant (*F*_(2,76)_ = 26.087, *P*<0.001, *η*^*2*^ = 0.407). The comparison showed that the mean amplitude of N400 was the largest in frontal region, followed by central region and parietal region in both groups (all *P*<0.05). There was no significant difference in the neurotic main effect or the interaction effect of neuroticism and brain region, as specified in Table 3.

**Table 3:** Mean amplitude comparison of N400 (M±SD, μv).

Neuroticism	Frontal region	Central region	Parietal region
Low neuroticism group	4.17 ± 7.04	3.37 ± 6.64	2.16 ± 6.04
High neuroticism group	5.68 ± 4.67	4.63 ± 4.22	3.85 ± 4.74

Note: Neurotic main effect (*F*_(1,38)_ = 0.527, *P* = 0.472, *η*^*2*^ = 0.014), main effect of the brain region (*F*_(2,76)_ = 26.087, *P*<0.001, *η*^*2*^ = 0.407); interaction effect of neuroticism and brain region (*F*_(2,76)_ = 0.140, *P* = 0.870, *η*^*2*^ = 0.004).

#### Latency of EEG component N400

The difference was not statistically significant in the neurotic main effect, main effect of the brain region or the interaction effect of neuroticism and brain region, as specified in Table 4.

**Table 4:** Comparison of the latency of N400 (M±SD, ms).

Neuroticism	Frontal region	Central region	Parietal region
Low neuroticism group	386.93 ± 54.05	412.10 ± 40.86	410.00 ± 54.15
High neuroticism group	373.88 ± 52.16	374.77 ± 42.90	391.85 ± 55.71

Note: Neurotic main effect (*F*_(1,38)_ = 0.024, *P* = 0.877, *η*^*2*^ = 0.014), main effect of the brain region (*F*_(2,76)_ = 2.136, *P* = 0.125, *η*^*2*^ = 0.407); interaction effect of neuroticism and brain region (*F*_(2,76)_ = 0.065, *P* = 0.938, *η*^*2*^ = 0.004).

#### Mean amplitude of EEG component LPC

The difference in main effect in the brain region was significantly different (*F*_(2,76)_ = 54.038, *P*<0.001, *η*^*2*^ = 0.587). The comparison showed that the mean amplitude of LPC was the largest in frontal region, followed by central region and parietal region successively in both groups (all *P*<0.05). There was no significant difference in the neurotic main effect or the interaction effect of neuroticism and brain region, as specified in Table 5.

**Table 5:** Mean amplitude comparison of LPC (M±SD, μv).

Neuroticism	Frontal region	Central region	Parietal region
Low neuroticism group	5.08 ± 6.59	3.53 ± 6.63	2.10 ± 6.30
High neuroticism group	6.26 ± 5.05	5.04 ± 4.87	4.17 ± 5.11

Note: Neurotic main effect (*F*_(1,38)_ = 0.890, *P* = 0.351, *η*^*2*^ = 0.023), main effect of the brain region (*F*_(2,76)_ = 54.038, *P*<0.001, *η*^*2*^ = 0.587); interaction effect of neuroticism and brain region (*F*_(2,76)_ = 1.866, *P* = 0.162, *η*^*2*^ = 0.047).

#### Latency of EEG component LPC

The difference in the main effect in the brain region was statistically significant (*F*_(2,76)_ = 3.876, *P* = 0.025, *η*^*2*^ = 0.587). The comparison showed that the latency of LPC in frontal region was shorter than that in central region (*P*<0.05). There was no significant difference in the neurotic main effect or the interaction effect of neuroticism and brain region, as specified in Table 6.

**Table 6:** Comparison of the latency of LPC (M±SD, ms).

Neuroticism	Frontal region	Central region	Parietal region
Low neuroticism group	639.37 ± 85.63	671.57 ± 81.30	682.73 ± 80.63
High neuroticism group	642.27 ± 89.04	671.83 ± 80.24	649.23 ± 80.36

Note: Neurotic main effect (*F*_(1,38)_ = 0.891, *P* = 0.351, *η*^*2*^ = 0.023), main effect of the brain region (*F*_(2,76)_ = 3.876, *P* = 0.025, *η*^*2*^ = 0.587); interaction effect of neuroticism and brain region (*F*_(2,76)_ = 1.293, *P* = 0.281, *η*^*2*^ = 0.047).

## Discussion

The results of the accuracy of DRM paradigm of false memory in this study showed that the accuracy of high neuroticism in senile people was significantly lower than that of low neuroticism in senile people, indicating that high neuroticism can increase false memory among senile people.

N400 is a negative ERPs component with a peak value about 400ms after stimulation [10], which usually appears at the electrode sites of the central and parietal regions, is closely related to the "familiarity" cognitive process in the memory recognition, and reflects the semantic processing. The study found that [11, 12] the amplitude of N400 was positively correlated with the semantic priming effect, and the latency represented the speed of semantic processing, that is, the larger the amplitude generally represents the higher the semantic priming effect, the more cognitive resources invested in semantic processing and the shorter the latency, the faster the semantic processing. It was found that for the mean amplitude of N400, the main effect of the brain region was significantly different, and the mean amplitude of N400 was the largest in frontal region, followed by central region and parietal region successively, suggesting that during semantic processing the semantic priming effect was the highest and the amount of cognitive resources invested was the most in frontal region, followed by central region and parietal region successively. There was no significant difference in the main effect of neuroticism, and the interaction effect between the two, suggesting that there was no obvious difference in the semantic priming effect and the amount of cognitive resources invested between the high neuroticism and low neuroticism during semantic processing. For the latency of N400, there was no significant difference in the neurotic main effect, the main effect of the brain region and the interaction effect between the two, suggesting that there was no obvious difference in the semantic processing speed between the high neuroticism and low neuroticism during semantic processing.

LPC is a late positive component with a peak value 500-800ms after stimulation [13–15], which is closely related to the “recollection” cognitive process during memory recognition and reflects the memory extraction process. The study found [16, 17] that the amplitude of LPC was positively correlated with the memory extraction effect, and the latency represented the speed of memory extraction, that is, the larger the amplitude generally represents the higher the memory extraction effect, the more cognitive resources invested in memory extraction and the shorter the latency, the faster the memory extraction. Research by Norman et al. [18] and Vaz et al. [19] consistently found that during the memory extraction stage, there was a close association between the special neurophysiological activity generated by the hippocampus (hippocampal sharp-wave ripples) and the brain cortex. This specific neurophysiological activity closely accompanies the reactivation and extraction of memory information in the brain cortex. In this study, it was found that there was a significant difference in the main effect of the brain region for the mean amplitude of LPC. The mean amplitude of LPC was the largest in frontal region, followed by central region and parietal region successively, suggesting that in the process of memory extraction, the memory extraction effect was the highest in frontal region, followed by central region and parietal region successively, the amount of cognitive resources consumed was the most in frontal region, followed by central region and parietal region successively; there was no significant difference in the neurotic main effect, suggesting that there was no significant difference in memory extraction effect and the amount of cognitive resources consumed between high neuroticism and low neuroticism during memory extraction. For the latency of LPC, There was no significant difference in the neurotic main effect and the interaction effect between the two, suggesting that there was no obvious difference in the speed of memory extraction between high neuroticism and low neuroticism in the process of memory extraction.

The study found that senile people with high neuroticism demonstrated significantly lower accuracy compared to senile people with low neuroticism. However, there were no significant difference in the components of N400 and LPC between the high and low neuroticism groups. This may be related to the gradual decline in brain function and decreased sensitivity of brain electrical activity in older individuals. We will further explore the impact of high neuroticism on semantic processing and memory extraction abilities by studying young people.

## Conclusions

High neuroticism can significantly increase the false memory of senile normal people. The EEG components of N400 and LPC are potential early indicators of high neuroticism affecting false memory. High neuroticism may influence false memory by affecting the frontal cortex function.

## Highlights

It was first to study false memory from the perspective of neuroticism. By analyzing the mean amplitude and latency of EEG components N400 and LPC and studying the differences in the brain region, it provides new thoughts for the study on high neuroticism affecting the ageing of false memory and ageing of cognitive function.

## Supporting information

S1 File (DOCX)

S2 File (PDF)

S1 Data (XLSX)

## References

[1] Anderson ND, Craik FI. 50 Years of Cognitive Aging Theory. J Gerontol B Psychol SciSoc Sci, 2017;72(1):1–6. doi: 10.1093/geronb/gbw108 . 27974471 PMC5156496

[2] Raji CA, Eyre H, Wei SH, et al. Hot Topics in Research: Preventive Neuroradiology in Brain Aging and Cognitive Decline. AJNR Am J Neuroradiol 2015;36(10):1803–1809. doi: 10.3174/ajnr.A4409 . 26045577 PMC4600413

[3] Devitt AL, Schacter DL. False memories with age: neural and cognitive underpinnings. Neuropsychologia,2016;91:346–359. doi: 10.1016/j.neuropsychologia.2016.08.030 . 27592332 PMC5075259

[4] Michelle JT, Anothai S, Corinne BN, et al. The influence of strategic encoding on false memory in patients with mild cognitive impairment and Alzheimer’s disease dementia. Brain Cogn, 2016;109:50–58. doi: 10.1016/j.bandc.2016.08.003 . 27643951 PMC5808440

[5] Katja V, Rudolf S, Dieter V, et al. Event-related potentials differ between true and false memories in the misinformation paradigm. Int J Psychophysiol, 2019;135:95–105. doi: 10.1016/j.ijpsycho.2018.12.002 . 30527597

[6] Kiat JE, Belli RF. An exploratory high-density EEG investigation of the misinformation effect: attentional and recollective differences between true and false perceptual memories. Neurobiol. Learn. Mem.2017;141:199–208. doi: 10.1016/j.nlm.2017.04.007 . 28442391

[7] Stadler MA; Roediger HL; McDermott KB. Norms for word lists that create false memories. MEM COGNITION. 1999;27(3):494–500. doi: 10.3758/bf03211543 . 10355238

[8] Curran T. Brain potentials of recollection and familiarity. Memory and Cognition, 2000, 28(6), 923–938. doi: 10.3758/bf03209340 . 11105518

[9] Delogu Francesca, Brouwer Harm, Crocker Matthew W. When components collide: Spatiotemporal overlap of the N400 and P600 in language comprehension. Brain Research. doi: 10.1016/j.brainres.2021.147514 . 33974906

[10] Katie H, Ludger H, Akira W, et al. The N400 indexes acquisition of novel emotion concepts via conceptual combination. Psychophysiology. 2021;58(2):e13727. doi: 10.1111/psyp.13727 . 33241553

[11] Tiedt HO, Ehlen F, Klostermann F. Age-related dissociation of N400 effect and lexical priming. Sci Rep. 2020;10(1):20291. doi: 10.1038/s41598-020-77116-9 . 33219241 PMC7680113

[12] Sora A, Se JO, Sang BJ, et al. Aging-Related Dissociation of Spatial and Temporal N400 in Sentence-Level Semantic Processing: Evidence From Source Analyses. Frontiers.2022;14:877235. doi: 10.3389/fnagi.2022.877235 . 35754967 PMC9226558

[13] Curran T, Cleary AM. Using ERPs to dissociate recollection from familiarity in picture recognition. Cogn Brain Res, 2003;15(2):191–205. doi: 10.1016/s0926-6410(02)00192-1 . 12429370

[14] Rugg MD, Curran T. Event-related potentials and recognition memory. Trends Cogn Sci, 2007;11(6): 251–257. doi: 10.1016/j.tics.2007.04.004 . 17481940

[15] Tsivilis D, Allan K, Roberts J, et al. Old-new ERP effects and remote memories: The late parietal effect is absent as recollection fails whereas the early mid-frontal effect persists as familiarity is retained. Front Hum Neurosci,2015;9:532. doi: 10.3389/fnhum.2015.00532 . 26528163 PMC4604239

[16] Shaghayegh S, Reza P, Azin K. FN400 and LPC Responses to Different Degrees of Sensory Involvement: A Study of Sentence Comprehension. Adv Cogn Psychol, 2020;16(1), 45–58. doi: 10.5709/acp-0283-6 . 32566053 PMC7293998

[17] Liang XL, Xiao F, Zhu YX, et al. How types of prior knowledge and task properties impact the category-based induction: diverging evidence from the P2, N400, and LPC effects. Biol Psychol,2020;156:107951. doi: 10.1016/j.biopsycho.2020.107951 . 32890634

[18] Norman Y, Yeagle EM, Khuvis S, Harel M, Mehta AD, Malach R. Hippocampal sharp-wave ripples linked to visual episodic recollection in humans. SCIENCE. 2019-08-16; 365 (6454). doi: 10.1126/science.aax1030 . 31416934

[19] Vaz AP, Inati SK, Brunel N, Zaghloul KA. Coupled ripple oscillations between the medial temporal lobe and neocortex retrieve human memory. SCIENCE. 2019-03-01; 363 (6430): 975–978. doi: 10.1126/science.aau8956 . 30819961 PMC6478623

