# Supplementary material for: Event-related potentials study on the effects of high neuroticism on senile false memory
Source: PLoS One. 2024 Aug 15;19(8):e0304646. doi: 10.1371/journal.pone.0304646 (PMC11326595; doi:10.1371/journal.pone.0304646)
Supplement: S1 File — (DOCX) [file pone.0304646.s001.docx]

**Word lists in the experiment**

1. Learned word lists in the experiment

*WINDOW: brightness, curtain, ventilation, sunshine, glass, sill, breathability, open, lightness and spaciousness, brightness, window grille, house, scenery

*BREAD: food, hungry, flour, milk, diet, cream, jelly, toast, biscuits, dough, breakfast, cheese

*GIRL: long hair, pigtail, long skirt, pretty, beautiful, boy, daughter, sister, elegant, dignified, cute

*SWEET: candy, sugar, honey, sugarcane, preserved fruit, milk candy, cake, date, dessert, bitter, sour, decayed tooth

*CHAIR: seat, stool, desk, sofa, desk, lazyback, sitting, bench, cushion, recliner, furniture, wood

*ANGER: annoyed, irritated, quarrel, hatred, enrage, anger, disgust, indignant, rebuke, criticize, rampage, hostility

*COLD: winter, ice and snow, frost, freeze, stove, shiver, snow, cotton jacket, ice-cold, warm, refrigerator, common cold
*DOCTOR: see a doctor, nurse, sick, cure, patient, injection, take medicine, recovery, patient, hospital, clinic, medicine
*MOUNTAIN: peak, steep, cliff, top, grand, precipitous, spectacular, climb, hummock, mountain climbing, hill, hillside

*RIVER: lake, stream, brook, swim, flow, bridge, boat, water plants, Yangtze River, sea, limpid, mountains and rivers

*MUSIC: art, melody, rhythm, dance, piano, sing, sound, play, band, note, musical composition, disc

*SLEEP: drowsy, rest, dream, yawn, snore, awake, pillow, bed, tired, sleepy, nap, night

2. Unlearned word lists in the experiment

*BLACK: white, charred, night, color, grief, blue, death, ink, bottom, coal, brown, gray

*CAR: truck, train, vehicle, drive, jeep, bus, game, key, garage, sedan, van, passenger car

*CITY: town, crowded, capital, streets, subway, country, Beijing, village, metropolis, Shanghai, suburb

*CUP: saucer, teacup, measuring, coaster, lid, handle, coffee, straw, goblet, drink, plastic, sip

*FRUIT: apple, vegetable, orange, citrus, ripe, pear, banana, berry, cherry, basket, juice, salad

*MAN: woman, husband, uncle, lady, male, father, strong, friend, beard, handsome, muscle, suit
*PEN: pencil, write, leak, quill, manuscript paper, scrawl, crayon, marker, nib, red, cap, letter

*ROUGH: smooth, bumpy, road, tough, sandpaper, coarse, riders, rugged, sand, boards, ground, gravel
*RUBBER: elastic, bounce, gloves, tire, tennis, resilient, rubber shoes, shoe soles, latex, glue, flexible, stretch

*SHIRT: blouse, sleeves, pants, tie, button, shorts, iron, collar, vest, pocket, belt, cuffs
*SLOW: fast, lethargic, stop, snail, cautious, delay, traffic, turtle, hesitant, speed, sluggish, wait

*SOFT: hard, light, pillow, plush, loud, cotton, fur, touch, fluffy, feather, kitten, skin
