# Supplementary material for: Event-related potentials study on the effects of high neuroticism on senile false memory
Source: PLoS One. 2024 Aug 15;19(8):e0304646. doi: 10.1371/journal.pone.0304646 (PMC11326595; doi:10.1371/journal.pone.0304646)
Supplement: S2 File — (PDF) [file pone.0304646.s002.pdf]

实验所用词表

1.实验所用学习词表

*窗户：明亮 窗帘 通风 阳光 玻璃 窗台 透气 打开 敞亮 窗花 房间 风景

*面包：食品 饥饿 面粉 牛奶 饮食 奶油 果酱 烘烤 饼干 面团 早餐 奶酪

*女孩：长发 辫子 长裙 漂亮 美丽 男孩 女儿 姐姐 妹妹 秀气 端庄 可爱

*甜味：糖果 白糖 蜂蜜 甘蔗 蜜饯 奶糖 蛋糕 红枣 点心 苦味 酸味 蛀牙

*椅子：座位 凳子 桌子 沙发 书桌 靠背 坐下 板凳 坐垫 躺椅 家具 木头

*愤怒：恼火 激怒 争吵 憎恨 发火 生气 厌恶 气愤 训斥 指责 暴跳 敌意

*寒冷：冬天 冰雪 霜冻 结冰 火炉 寒颤 雪花 棉袄 冰冷 暖和 冰箱 感冒

*医生：看病 护士 生病 治疗 病人 打针 吃药 康复 患者 医院 诊所 医药

*高山：顶峰 陡峭 悬崖 高峰 雄伟 险峻 壮观 攀登 山岗 登山 丘陵 山坡

*河流：湖泊 河水 溪流 游泳 流淌 桥梁 小船 水草 长江 大海 清澈 山川

*音乐：艺术 旋律 节拍 舞蹈 钢琴 歌唱 动听 演奏 乐队 音符 乐曲 唱片

*睡觉：犯困 休息 做梦 呵欠 打鼾 睡醒 枕头 床铺 疲倦 瞌睡 打盹 夜晚

2.实验所用未学词表

*黑色：白色 烧焦 夜晚 颜色 悲伤 蓝色 死亡 墨水 底部 煤炭 棕色 灰色

*汽车：卡车 火车 车辆 驾驶 吉普 公交 比赛 钥匙 车库 轿车 货车 客车

*城市：城镇 拥挤 县城 首都 街道 地铁 国家 北京 村庄 都市 上海 郊区

*杯子：碟子 茶杯 测量 杯垫 盖子 把手 咖啡 吸管 酒杯 饮料 塑料 酌饮

*水果：苹果 蔬菜 橙子 柑橘 成熟 梨子 香蕉 浆果 樱桃 篮子 果汁 沙拉

*男人：女人 丈夫 叔叔 女士 男性 父亲 强壮 朋友 胡子 英俊 肌肉 西装

*钢笔：铅笔 书写 漏墨 毛笔 稿纸 涂鸦 蜡笔 记号 笔尖 红色 笔帽 字母

*粗糙：光滑 颠簸 道路 坚韧 砂纸 粗织 骑手 崎岖 沙子 木板 地面 砾石

*橡胶：弹性 弹跳 手套 轮胎 网球 弹性 胶鞋 鞋底 乳胶 胶水 柔性 拉伸

*衬衫：上衣 袖子 裤子 领带 纽扣 短裤 熨斗 领子 背心 口袋 腰带 袖口

*缓慢：快速 昏睡 停止 蜗牛 谨慎 延迟 交通 乌龟 犹豫 速度 呆滞 等待

*柔软：坚硬 轻柔 枕头 毛绒 响亮 棉花 毛皮 触感 蓬松 羽毛 小猫 皮肤
